# AS01_B_-, MF59-, and alum-based adjuvants and HIV vaccine immunogenicity: a *post-hoc* cross-protocol comparison of results from HVTN 100, 107, 120, and 702

**DOI:** 10.3389/fimmu.2026.1768201

**Published:** 2026-03-12

**Authors:** Ferdinando Menezes, Shiyu Chen, Nicole Grunenberg, Mookho Malahleha, Kathy Mngadi, Monde Muyoyeta, Fatima Laher, Zvavahera Mike Chirenje, Linda-Gail Bekker, Paul Goepfert, Glenda E. Gray, Georgia Tomaras, M Juliana McElrath, Esper Kallas, Alison C. Roxby, Zoe Moodie

**Affiliations:** 1 Centro de Pesquisas Clinicas, University of Sao Paulo, Sao Paulo, Brazil; 2 Vaccine and Infectious Disease Division, Fred Hutchinson Cancer Center, Seattle, WA, United States; 3 Setshaba Research Centre, University of Cape Town, Cape Town, Soshanguve, South Africa; 4 Aurum Institute, Tembisa, Gauteng, South Africa; 5 Centre for Infectious Diseases Research in Zambia, Livingstone, Zambia; 6 Perinatal HIV Research Unit, Faculty of Health Sciences, University of the Witwatersrand, Johannesburg, South Africa; 7 Department of Obstetrics and Gynecology, University of California San Francisco, San Francisco, CA, United States; 8 Faculty of Medicine and Health Science, University of Zimbabwe Clinical Trials Research Centre, University of Zimbabwe, Harare, Zimbabwe; 9 Desmond Tutu HIV Centre, University of Cape Town, Cape Town, South Africa; 10 Department of Medicine, University of Alabama at Birmingham, Birmingham, AL, United States; 11 South African Medical Research Council, Cape Town, South Africa; 12 Center for Human Systems Immunology, Duke University School of Medicine, Durham, NC, United States; 13 Department of Surgery, Duke University Medical Center, Durham, NC, United States; 14 Cape Town HIV Vaccine Trials Network Immunology Laboratory, Cape Town, South Africa

**Keywords:** adjuvant, alum, AS01B, HIV, MF59, vaccine

## Abstract

**Introduction:**

Vaccine adjuvants are crucial in HIV vaccine development, enhancing immune responses. Immune responses generated by different adjuvanted vaccines are rarely compared directly with the same vaccine antigens.

**Methods:**

This retrospective cross-protocol, cross-sectional analysis examined four randomized, controlled, double-blinded, multicenter trials (HVTN 100, 107, 120, and 702) of adults without HIV who received the ALVAC-HIV (vCP2438) vaccine and a bivalent gp120 protein booster with either MF59, Alum, or AS01_B_ adjuvant. Participants received ALVAC-HIV at months 0, 1, 3, 6, and 12, and the adjuvanted protein at months 3, 6, and 12. Data from month 6.5 were compared for IgG V1V2 binding antibodies (bAb) and CD4+ T-cell responses, measured as IFN-γ and/or IL-2 expression, using Wilcoxon and Barnard’s tests with FDR p-value multiplicity adjustment. Reactogenicity and adverse event profiles were also assessed.

**Results:**

AS01_B_ elicited higher CD4+ T cell magnitudes among positive responders than either MF59 or Alum adjuvant to the 3 antigens considered: 1086, TV1, ZM96. No statistically significant differences were observed between MF59 and Alum in CD4+ T-cell responses. Regarding IgG bAb responses, AS01_B_ induced significantly higher magnitudes among positive responders compared to both MF59 and Alum for the C.1086C V1V2 and Con 6 gp120/B antigens. Additionally, AS01_B_ elicited greater IgG bAb responses than MF59 for gp70-96ZM651.02 V1V2 and gp70_B.CaseA V1V2, although no significant differences were found between AS01_B_ and Alum for these antigens. AS01_B_ also showed a trend toward greater reactogenicity, though this difference did not reach statistical significance. Importantly, no serious adverse events occurred in any of the groups.

**Conclusion:**

The AS01_B_ adjuvant demonstrated superior IgG binding antibody and CD4+ T-cell responses compared with MF59 and Alum, when given with gp120 protein boost after ALVAC-HIV prime. These findings support AS01_B_ as a superior adjuvant for HIV vaccine development, relative to MF59 and Alum.

## 1 Introduction

Vaccine adjuvants are essential components in modern immunization strategies, playing a critical role in enhancing immune responses while reducing the antigen dose required for effective protection. In the case of preventive HIV vaccines, generating a strong antibody response alongside a robust T cell response is crucial. Adjuvants initiate a targeted response by activating innate immune pathways that sometimes lead to the improvement of the subsequent adaptive response. Their ability to modulate antigen presentation through dendritic cell activation and cytokine upregulation is particularly valuable in addressing the challenges posed by the HIV virus’s high mutation rate and complex pathogenesis.

Among the diverse adjuvants employed, AS01_B_, MF59, and Alum each exhibit unique mechanisms of action. AS01_B_, a liposome-based formulation, incorporates monophosphoryl lipid A (MPL), a detoxified derivative of bacterial lipopolysaccharide that signals via Toll-like receptor 4 (TLR4) and QS-21, a saponin that significantly boosts antigen-antibody responses (1, 2). In contrast, MF59, an oil-in-water emulsion, enhances immune responses by recruiting antigen-presenting cells and upregulating a broad spectrum of cytokines and chemokines (2–4). Alum, with its well-documented depot effect, prolongs antigen availability and enhances humoral immunity (2, 3, 5, 6). These distinct mechanisms result in the potential to elicit varying degrees of immunogenicity.

A major challenge in vaccine development is finding the right balance between a potent immune response and minimizing adverse reactions. While strong adjuvants can significantly boost immunity, they may also heighten solicited local and systemic reactions such as injection-site pain or fever (7, 8). Parallel assessments of reactogenicity alongside specific immune responses are needed to fully describe the effects of adjuvants.

Although numerous studies have evaluated these adjuvants individually (9–15), interpretation of their relative performance has often been limited by differences in immunogens, vaccination schedules, assay platforms and analytical approaches across trials. Prospective head-to-head comparisons have been conducted within individual HIV vaccine studies. In HVTN 107 (16), MF59 and Alum were directly compared and demonstrated comparable cellular and humoral immune responses. In contrast, HVTN 120 (17) directly compared MF59 and AS01_B_ and reported superior immune responses with AS01_B_. However, these comparisons were restricted to pairwise evaluations within individual trial contexts and did not allow simultaneous assessment of all three adjuvants under harmonized analytical conditions.

The present study leverages data from four preventive HIV vaccine trials (HVTN 100, 107, 120, and 702) (16–19) that employed a common ALVAC-HIV prime and bivalent subtype C gp120 protein boost, with identical antigen doses, vaccination schedules, and standardized immunogenicity timepoints, differing primarily in the adjuvant formulation. By applying a unified statistical and analytical framework to these harmonized datasets, this cross-protocol analysis enables a direct comparative evaluation of AS01B, MF59, and Alum with respect to CD4^+^ T-cell responses, IgG binding antibody magnitudes, and reactogenicity. This integrative approach provides insights into adjuvant-specific effects that could not be derived from individual trial reports alone.

## 2 Materials and methods

This was a retrospective, cross-protocol, cross-sectional analysis of the four randomized, controlled, double blinded, multicenter trials: HVTN 100 (NCT02404311), HVTN 107 (NCT03284710), HVTN 120 (NCT03122223) and HVTN 702 (NCT02968849). The four trials each enrolled healthy adults without HIV between the ages of 18 and 40 years old.

All trials evaluated an ALVAC-HIV vector-based vaccine (vCP2438) with an insert expressing an envelope glycoprotein subtype C gp-120 (derived from HIV-1 96ZM651) linked to the transmembrane anchor sequence of gp-41, gag and protease (all three from HIV-1 B Lymphadenopathy-Associated Virus isolate - LAI). The booster consisted of bivalent subtype C gp-120 envelope proteins (TV1.C and 1086.C), with 100µg of each recombinant protein combined with the respective adjuvant. The gp120 proteins were co-formulated with the assigned adjuvant according to the parent trial manufacturing specifications and administered as a single intramuscular injection per protocol design (16–19).

All immunogenicity data analyzed in this study were generated prospectively as part of the original HVTN 100, 107, 120, and 702 clinical trial protocols and were not newly produced for the present cross-protocol analysis. BAMA and intracellular cytokine staining (ICS) assays were conducted according to pre-specified, validated protocols in centralized GCLP-compliant laboratories. The present study constitutes a retrospective, harmonized reanalysis of these previously generated datasets using a unified statistical framework to enable direct comparisons of adjuvant-specific immune responses.

In all four trials, participants received ALVAC-HIV injections at months 0, 1, 3, 6, 12 and adjuvanted gp120 protein booster vaccines at months 3, 6, 12. This analysis used data from a single time point, month 6.5, which occurred 2 weeks after the second protein booster dose. The month 6.5 timepoint was selected because it corresponds to the expected peak of vaccine-induced immune responses following the second protein boost and allows for standardized comparison of adjuvant-specific effects across trials.

CD4^+^ T-cell responses were measured by intracellular cytokine staining (ICS), and IgG V1V2 binding antibodies were assessed using the binding antibody multiplex assay (BAMA) against vaccine-matched antigens 1086, ZM96, and TV1. To assess heterologous responses, IgG binding antibodies to the consensus antigen Con 6 gp120/B were also measured. The BAMA readout was obtained as the background-subtracted mean fluorescence intensity (MFI), where the background included both an antigen-specific plate level control (i.e. a blank well containing antigen-coated beads run on each plate) and a specimen-specific control (i.e. a serum well containing blank beads). Samples were considered positive if they met the following criteria: (1) net MFI ≥ antigen-specific positive response threshold (defined separately for each trial as the maximum of 100 and the 95th percentile of pre-vaccination/baseline net MFI values), (2) net MFI > 3 times the baseline net MFI, and (3) MFI > 3 times the baseline MFI. IgG responses were assayed at a dilution of 1:50 (20).

IgG3 binding antibody responses were assessed in selected parent trials; however, these data were available only for MF59 and Alum recipients and were not collected for the AS01B cohort. Because the present study aimed to perform a harmonized three-way comparison across adjuvants, IgG3 responses were not included in the comparative analysis. ICS results were measured using a validated flow cytometry assay (21, 22). Cryopreserved PBMCs were stimulated with synthetic HIV-1 Envelope peptide pools. Dimethyl Sulfoxide (DMSO), the diluent for the peptide pools, served as the negative control (run in duplicate), while cells stimulated with staphylococcal enterotoxin B (SEB), a polyclonal stimulant, served as the positive control. The background-adjusted percentage of CD4 + and CD8+ T cells expressing IFN-γ and/or IL-2 was analyzed, where this net percent was calculated as % of antigen-stimulated cells minus % of unstimulated, negative control cells. A positive response was determined based on a one-sided Fisher’s exact test, comparing the frequency of IFN-γ and/or IL-2-producing cells in the peptide-stimulated well to that in the negative control wells (20).

All BAMA assays were performed in the Tomaras lab (Duke University). ICS assays were run by the HVTN Laboratory Center in either the Seattle (Fred Hutchinson Cancer Center) or the Cape Town (Cape Town HVTN Immunology Laboratory) laboratory; assay concordance has been demonstrated between the two laboratories. No additional laboratory testing was performed for this cross-protocol analysis; all comparisons used existing quality-controlled datasets generated within the original trials.

All p-values are two-sided, with unadjusted p-values less than 0.05 and False Discovery Rate (FDR)-adjusted p-values (*p) < 0.10 deemed statistically significant. FDR adjusted p-values were reported to account for the multiple testing across antigens in the pairwise comparisons of adjuvants. Wilcoxon tests were used to compare magnitude of responses among positive responders for pairwise comparisons of MF59 vs. Alum vs. AS01_B_ for both IgG binding antibody and CD4+ T cell responses. Barnard’s tests were used to compare positive response rates for pairwise comparisons of MF59 vs. Alum vs. AS01_B_ for both IgG binding antibody and CD4+ T cell responses.

Immunogenicity analyses included per-protocol participants (PP), those who were not diagnosed with HIV-1 and who received all scheduled vaccinations prior to the primary month 6.5 immunogenicity blood draw. Samples that failed assay-specific quality-control criteria were excluded.

Reactogenicity data were collected using standardized questionnaires. There was also a comparison of reactogenicity, and product-related adverse events reported among participants receiving different adjuvants. Reactogenicity data was available for the modified intent-to-treat (MITT) participants, resulting in a larger sample than used for the immunogenicity analyses. For this data, the maximum severity for each symptom is the highest severity experienced over all the vaccinations received by the participant, if the onset date was within 7 days following vaccination.

All statistical analyses were conducted using R (version 4.0.4, R Foundation for Statistical Computing, Vienna, Austria) and SAS (version 9.4, SAS Institute, Cary, North Carolina, USA).

All protocols were approved by the Fred Hutchinson Cancer Center institutional review board (IRB), and local research ethics committees for each site. All participants gave written informed consent in the language of their choice prior to enrollment.

## 3 Results

### 3.1 Characteristics of the study population

From the four included trials, HVTN 100, 107, 120 and 702, a total of 3,000 participants received the MF59-based adjuvant, 36 received the alum-based adjuvant, and 50 received the AS01_B_-based adjuvant. Because immunogenicity assessments were only done on a randomly selected subset of participants in HVTN 702 as described in *Moodie* et al. (23), we included available data from 60 participants in HVTN 702 who received MF59, for a total of 320 participants in the MF59 group, 27 in the Alum group, and 46 in the AS01_B_ group (**Table 1**).

**Table 1:** Study populations of the four clinical trials of ALVAC-HIV and Bivalent Subtype C gp120, and number of participants in each analysis.

Study	Adjuvant	Number of participants (PP): immunogenicity (N)	Number of participants (MITT): reactogenicity (N)
HVTN 100	MF59	185	210
HVTN 120	MF59	48	50
HVTN 120	AS01_B_	46	50
HVTN 107	MF59	27	36
HVTN 107	Alum	27	36
HVTN 702	MF59	60	2704

PP, Per-protocol; MITT, modified intent to treat; N, number of participants.

The three groups had similar demographic characteristics: 56.5% were female, with a median age of 24 years, a median body mass index (BMI) of 22.8, and most were from South Africa (70.5%). Detailed demographic data by protocol are provided in the **Supplementary Material** (**Supplementary Tables 1**-**2**). Reactogenicity data were collected for all participants that received at least one dose of the vaccine, either with the prime or the boost product, in each of the trials.

### 3.2 Immunogenicity data

#### 3.2.1 CD4+ T cell responses

For the intracellular cytokine staining (ICS) CD4^+^ T-cell responses, we measured IFN-γ and/or IL-2 expression in response to three antigens: 1086 gp120, TV1 gp120, and ZM96 gp120 (**Table 2**).

**Table 2:** Antigen-specific T-cell response rates, comparing adjuvants using Barnard tests to compare response rates for pairwise comparisons of AS01_B_ vs. MF59 or ALUM. FDR-adjusted p-values are reported.

ICS CD4+ T-cell response rates by antigen and adjuvant					
Antigen	MF59 (%)	Alum (%)	AS01_B_ (%)	Adj. p-value (MF59 vs AS01_B_)	Adj. p-value (Alum vs AS01_B_)
1086 gp120	46.8	62.5	90.0	< 0.001	0.018
TV1 gp120	62.6	70.8	95.0	0.004	0.016
ZM96 gp120	55.1	62.5	87.5	0.006	0.032

**Figure 1:**
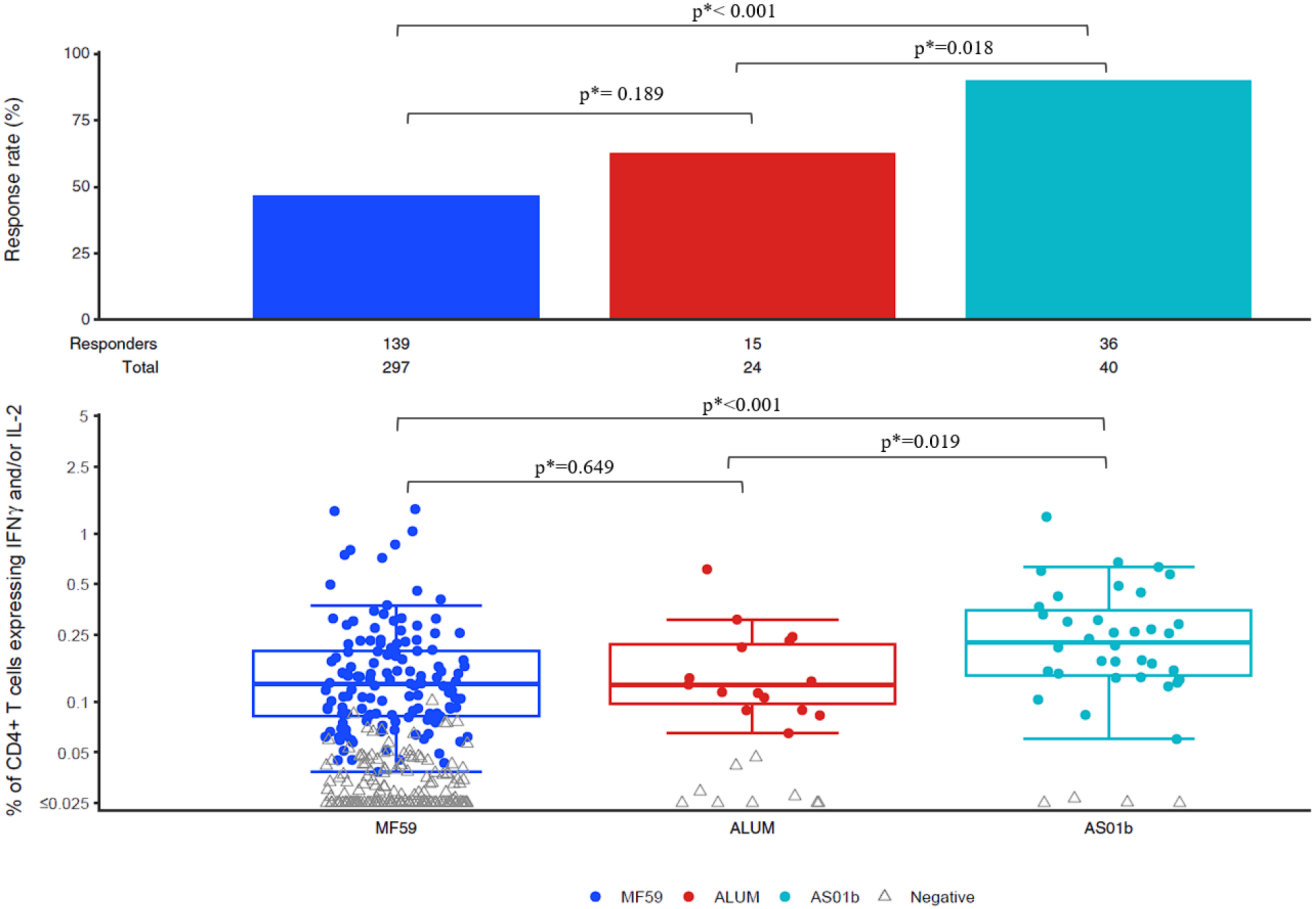
Intracellular cytokine staining (ICS)T-cell assays: Response rates and magnitudes of CD4+ IFNy and/or IL-2 Expression in response to 1086 gp120.

Vaccine regimens that included a protein boost adjuvanted with AS01_B_ consistently induced significantly higher magnitudes of CD4^+^ T-cell responses among positive responders compared to MF59 for all three antigens. Specifically, for 1086 gp120 (**Figure 1**), the difference was significant (p < 0.001; p*<0.001). Similarly, AS01_B_ elicited significantly higher responses to TV1 gp120 (p < 0.001; p*<0.001) and ZM96 gp120 (p = 0.005; p*=0.015).

In comparisons with Alum, AS01_B_ adjuvanted regimens also showed higher response magnitudes among positive responders across all 3 antigens. These differences were statistically significant for 1086 gp120 (p = 0.008; p*=0.019) and ZM96 gp120 (p = 0.022; p*=0.040). However, the difference for TV1 gp120 did not reach statistical significance (p = 0.073; p*=0.109) despite a consistent trend favoring AS01_B_ (**Figures 1**, **Supplementary Figures S1**-**2**).

No statistical differences were observed between MF59 and Alum in any of the ICS comparisons.

#### 3.2.2 IgG binding antibody responses

**Figure 2:**
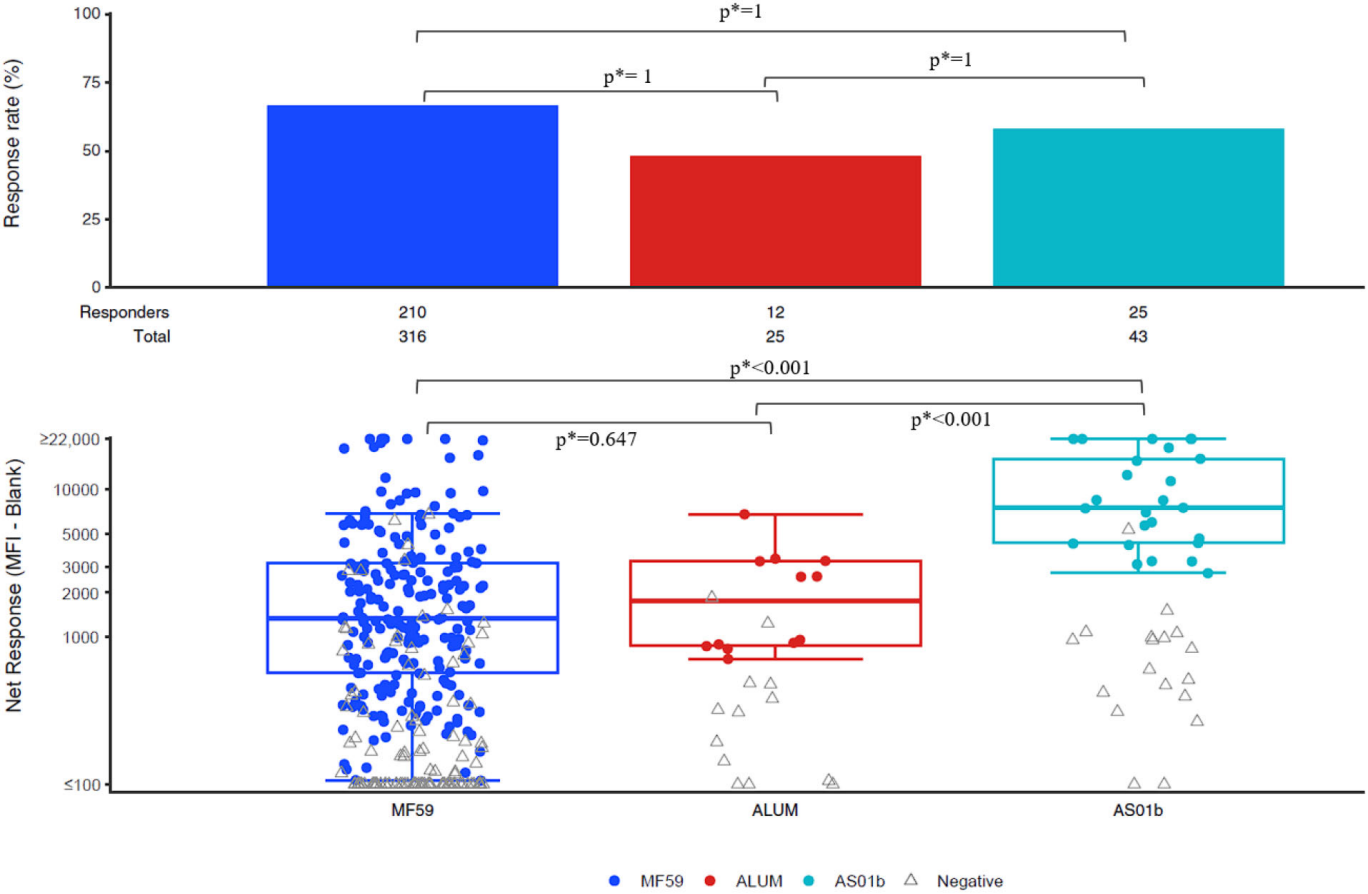
IgG Binding Antibody Response rates and magnitudes to C.1086C_V1_V2 Tags (Dilution 1:50).

There were no significant differences in IgG response rates between adjuvanted vaccine regimens for any of the antigens analyzed: C.1086C_V1_V2 Tags; gp70-TV1.GSKvacV1V2/293F; gp70-96ZM651.02 V1V2; AE.A244 V1V2 Tags/293F; gp70_B.CaseA_V1V2; Con 6 gp120/B (**Figure 2**).

Vaccine regimens with the AS01_B_ adjuvant resulted in significantly higher IgG magnitudes among positive responders against both C.1086C_V1V2 and Con 6 gp120/B antigens, when compared to regimens using MF59 and Alum. For the C.1086C_V1V2 antigen, the median response was 1,340.6 in the MF59 group and 7,514.8 in the AS01_B_ group (p < 0.001; p*<0.001), and the median response for Alum was 1,756 vs 7,514.8 for AS01_B_ (p < 0.001; p*<0.001). Similar significant differences showing AS01_B_ superiority were seen for the Con 6 gp 120/B antigen (both p <0.001; p*<0.001) (**Supplementary Figure S3**).

For the gp70-96ZM651.02 V1V2 and gp70_B.CaseA_V1V2 antigens, AS01_B_ elicited higher responses among positive responders than MF59 (p < 0.001; p*<0.001 and p=0.01; p*=0.026, respectively). However, no significant differences were observed when compared to Alum for these two antigens. For the gp70-TV1.GSKvacV1V2/293F antigen there were no differences in magnitudes among positive responders between responses for the three adjuvants. There were no statistically significant differences in the magnitude of IgG responses among positive responders between MF59 and Alum for any antigen evaluated (**Supplementary Figures 4**-**7**).

### 3.3 Reactogenicity

**Figure 3:**
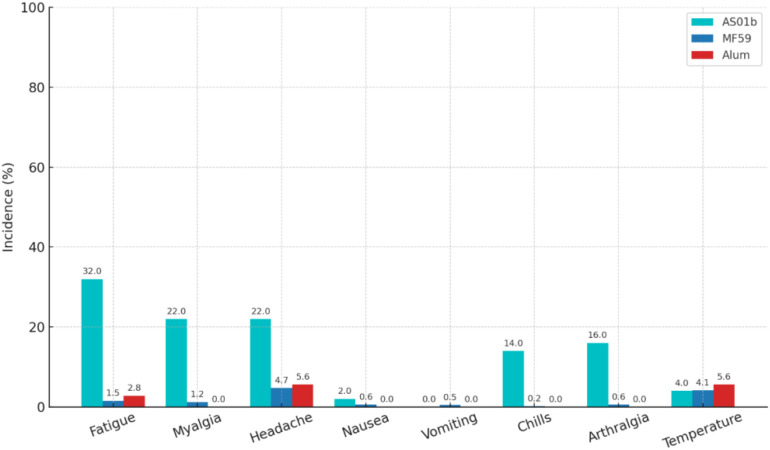
Moderate or severe systemic adverse events across three trials of ALVAC-HIV with Bivalent Subtype C gp120, comparing AS01_B_, MF 59, and Alum adjuvants.

Reactogenicity data were analyzed from all MITT participants in the 4 trials where 3000 participants received MF59, 36 received alum, and 50 received AS01_B_-based adjuvant (**Table 1**). All adjuvanted vaccine regimens demonstrated a favorable safety profile. The most common local effects for all products were mild pain and tenderness. However, AS01_B_ adjuvanted regimens were associated with a higher frequency of moderate local tenderness and a few moderate to severe systemic side effects, including fatigue, myalgia, headache, chills, and arthralgia, compared to the other adjuvanted regimens (**Figure 3**).

Detailed reactogenicity data are provided in **Supplementary Tables 3**-**4**.

## 4 Discussion

In this harmonized cross-protocol analysis of four preventive HIV vaccine trials using an identical immunogen and vaccination schedule, we demonstrate that the AS01B adjuvant consistently elicits greater CD4^+^ T-cell response magnitudes and higher IgG binding antibody levels among positive responders compared with MF59 and Alum. The primary contribution of this study lies in enabling a simultaneous, three-way comparison of adjuvant effects under standardized immunological and analytical conditions, thereby isolating the contribution of the adjuvant itself while minimizing confounding introduced by inter-trial heterogeneity.

The findings of the present cross-protocol analysis are consistent with prior prospective head-to-head comparisons conducted within individual HVTN trials. The absence of meaningful differences between MF59 and Alum observed here aligns with results reported in HVTN 107 (16), while the superior immunogenicity associated with AS01B relative to MF59 mirrors findings from HVTN 120 (17). By integrating data across multiple trials within a unified analytical approach, the current study places these individual findings into a broader comparative context.

Unlike the original prospective trial publications, which evaluated adjuvants in isolation, this study distinguishes between response rates and response magnitudes among positive responders and applies a consistent statistical framework across multiple antigens. This distinction is particularly relevant for vaccine design, as magnitude of response among responders may better reflect qualitative differences in immune priming and expansion than responder frequency alone.

Furthermore, the parallel evaluation of immunogenicity and reactogenicity across adjuvants provides a translational perspective on the balance between immune potency and tolerability, an aspect that cannot be readily assessed through single-trial analyses.

Binding antibody analyses by BAMA further supported the superiority of AS01_B_, especially against the C.1086C_V1V2 and Con 6 gp120/B antigens. Although some antigens such as gp70-TV1.GSKvacV1V2/293F showed no differential response between adjuvants, the overall trend favored AS01_B_. These findings align with data suggesting that AS01_B_, through its combination of MPLA (TLR4 agonist) and QS-21, induces a more robust activation of antigen-presenting cells and broader cytokine responses than oil-in-water emulsions like MF59 or mineral salt adjuvants like Alum (1, 25–27). This combination creates a synergistic effect, where MPLA drives cytokine production and dendritic cell activation, while QS-21 enhances antigen presentation and CD4+ T cell differentiation. The liposomal carrier not only facilitates co-localization of both molecules at the site of immune activation but also ensures controlled release and enhanced uptake by antigen-presenting cells, amplifying the innate-to-adaptive immune transition (1).

While AS01_B_’s heightened immunogenicity is a favorable attribute, it was also associated with increased reactogenicity, including systemic symptoms such as fatigue and myalgia. Despite this, the absence of severe adverse events confirms its acceptable safety profile. These observations reflect a well-documented trade-off between immunogenic potency and tolerability observed in other vaccine platforms using AS01_B_, such as the Shingrix vaccine (24).

The trials used standardized designs, case report forms, and harmonized immunogenicity endpoints, with the specimens analyzed here collected at a common time point (2 weeks after the primary series HIV immunization), and assayed in centralized laboratories using standardized, validated assays. A single Statistical and Data Management Center was responsible for all data cleaning and analysis. Another key strength of this analysis is its comparative nature across trials with harmonized vaccine platforms (ALVAC + bivalent subtype C gp120) but differing adjuvants, which is scarce in the literature, especially in the field of preventive HIV vaccine design. The consistency of these findings with prior individual trial reports further supports the robustness and generalizability of the observed adjuvant-specific effects.

Nevertheless, this study has limitations. Sample sizes differed across adjuvant groups, reflecting the original design and scale of the parent trials. Although non-parametric statistical methods were used to mitigate the impact of this imbalance, smaller groups may have reduced power to detect modest differences.

In addition, the present analysis focused on the Month 6.5 peak immunogenicity timepoint. Although extended follow-up was included in the parent trials, later timepoints were not uniformly available and/or were not assessed using identical antigen panels across protocols, limiting the validity of cross-protocol durability comparisons. Moreover, durability reflects distinct immunological processes, including the maintenance of long-lived plasma cells and memory B-cell populations, which are not directly inferred from peak response magnitude. Dedicated durability-focused analyses will be required to determine whether the enhanced peak responses observed with AS01B translate into sustained immunity.

This analysis was restricted to immune endpoints that were uniformly collected across all three adjuvant groups. Although IgG3 responses were measured in selected trials, these data were not available for the AS01B cohort and therefore could not be incorporated into the harmonized cross-protocol comparison. In addition, other IgG subclasses and Fc-mediated functional assays were not uniformly performed across all protocols. Future studies designed with integrated subclass and functional profiling across adjuvant groups will be important to further define qualitative differences in vaccine-induced immunity.

In summary, our study reinforces the immunological advantage conferred by AS01_B_ in HIV vaccine formulations. These findings provide a compelling rationale for its continued inclusion in future preventive HIV vaccine regimens and support its further investigation in combination strategies aiming for both potent immunogenicity and acceptable tolerability profiles.

The insights gained from this comparative analysis could also be used as rationale-leveraging experiments using other TLR agonists beyond MPLA to determine whether tailored adjuvant combinations could further optimize the balance between immunogenicity, durability, and reactogenicity in next-generation preventive HIV vaccine candidates.

## Data Availability

The raw data supporting the conclusions of this article will be made available by the authors, without undue reservation.
