# Supplementary material for: AS01B-, MF59-, and alum-based adjuvants and HIV vaccine immunogenicity: a post-hoc cross-protocol comparison of results from HVTN 100, 107, 120, and 702
Source: Front Immunol. 2026 Mar 12;17:1768201. doi: 10.3389/fimmu.2026.1768201 (PMC13017943; doi:10.3389/fimmu.2026.1768201)
Supplement: Supplementary file 1 [file Table1.docx]

Supplementary Material

# Tables

| Characteristics | MF59 | ALUM | AS01b | Total |
| --- | --- | --- | --- | --- |
| **N** |  |  |  |  |
| n | 320 | 27 | 46 | 393 |
| **Age** |  |  |  |  |
| Median (25-75) | 24 (21 – 27) | 25 (21.5 – 27) | 23.5 (21.2 – 27) | 24 (21 – 27) |
| **Sex (%)** |  |  |  |  |
| Female | 178 (55.6) | 17 (63.0) | 27 (58.7) | 222 (56.5) |
| Male | 142 (44.3) | 10 (37.0) | 19 (41.3) | 171 (43.5) |
| **Gender (%)** |  |  |  |  |
| Female | 171 (53.4) | 13 (48.1) | 25 (54.3) | 209 (53.2) |
| Male | 138 (43.1) | 7 (25.9) | 17 (37) | 162 (41.2) |
| Gender Diverse | 3 (0.93) |  | 4 (8.7) | 7 (1.8) |
| Not Known | 8 (2.5) | 7 (25.9) |  | 15 (3.8) |
| **BMI** |  |  |  |  |
| Median (25-75) | 23.7 (20 – 31.2) | 22.2 (20.4 – 27.8) | 22 (20.4 – 26.1) | 22.8 (20.5 – 27.2) |
| **Country (%)** |  |  |  |  |
| Mozambique | 3 (0.93) | 4 (14.8) |  | 7 (1.8) |
| South Africa | 261 (81.5) | 16 (59.3) |  | 277 (70.5) |
| Tanzania | 14 (4.37) |  | 14 (30.4) | 28 (7.1) |
| United States | 19 (5.94) |  | 17 (37) | 36 (9.2) |
| Zambia | 5 (1.56) |  | 5 (10.9) | 10 (2.5) |
| Zimbabwe | 18 (5.62) | 7 (25.9) | 10 (21.7) | 35 (8.9) |

Table S1 -Baseline demographics for participants with immunogenicity data.

| Characteristics | MF59 | ALUM | AS01b | Total |
| --- | --- | --- | --- | --- |
| **N** |  |  |  |  |
| n | 3000 | 36 | 50 | 3086 |
| **Age** |  |  |  |  |
| Median (25-75) | 24 (21 – 28) | 25 (21.8 – 27) | 23.5 (21 – 27.8) | 24 (21 – 28) |
| **Sex (%)** |  |  |  |  |
| Female | 2035 (67.8) | 21 (58.3) | 30 (60) | 2086 (67.6) |
| Male | 965 (32.2) | 15 (41.7) | 20 (40) | 1000 (32.4) |
| **Gender (%)** |  |  |  |  |
| Female | 2032 (67.7) | 17 (47.2) | 27 (54) | 2076 (67.3) |
| Male | 952 (31.7) | 11 (30.6) | 18 (36) | 981 (31.8) |
| Gender Diverse | 8 (0.2) |  | 5 (10) | 13 (0.4) |
| Not Known | 8 (0.2) | 8 (22.2) |  | 16 (0.5) |
| **BMI** |  |  |  |  |
| Median (25-75) | 24 (21 – 29) | 22.2 (20.2 – 26.7) | 22.3 (20.5 – 26.4) | 24.2 (20.9 – 29.5) |
| **Country (%)** |  |  |  |  |
| Mozambique | 6 (0) | 7 (19.4) |  | 13 (0.4) |
| South Africa | 2936 (97.8) | 21 (58.3) |  | 2957 (95.8) |
| Tanzania | 15 (0.5) |  | 15 (30) | 30 (1.0) |
| United States | 20 (0.6) |  | 20 (40) | 40 (1.3) |
| Zambia | 5 (0) |  | 5 (10) | 10 (0.3) |
| Zimbabwe | 18 (0.6) | 8 (22.2) | 10 (20) | 36 (1.2) |

Table S2 - Baseline demographics for participants with reactogenicity data.

| LOCAL REACTOGENICITY | HVTN 120 (MF59)  (n=50) | HVTN 107 (MF59)  (n=36) | HVTN 100  (MF59)  (n=210) | HVTN 702  (MF59)  (n=2704) | HVTN 107 (Alum)  (n=36) | HVTN 120 (AS01b)  (n=50) |
| --- | --- | --- | --- | --- | --- | --- |
| Pain (%)  None  Mild  Moderate  Severe | 16 (32)  27 (54)  6 (12)  1 (2) | 21 (58.3)  14 (38.9)  1 (2.8)  0 | 37 (17.6)  120 (57.1)  50 (23.8)  3 (1.4) | 2166 (80.1)  476 (17.6)  61 (2.3)  1 (0) | 20 (55.5)  13 (36.2)  3 (8.3)  0 | 18 (36)  17 (34)  13 (26)  2 (4) |
| Tenderness (%)  None  Mild  Moderate  Severe | 21 (42)  22 (44)  6 (12)  1 (2) | 20 (55.6)  16 (44.4)  0  0 | 60 (28.6)  114 (54.3)  35 (16.7)  1 (0.5) | 2379 (88)  289 (10.7)  35 (1.3)  1 (0) | 20 (55.5)  15 (41.8)  1 (2.7)  0 | 20 (40)  12 (24)  17 (34)  1 (2) |
| Erythema (%)  None  >6.25 to <25 cm^2^ area  25 to 100 cm^2^ area  >= 100 cm^2^ area | 41 (82)  1 (2) 2 (4)  0 | 30 (83.3)  1 (2.8)  0  0 | 183 (87.1)  7 (3.3)  2 (1)  2 (1) | 2494 (92.2)  108 (4)  74 (2.7)  28 (1) | 31 (86)  0  0  0 | 35 (70) 1 (2)  2 (4)  3 (6) |
| Induration (%)  None  >6.25 to <25 cm^2^ area  25 to 100 cm^2^ area  >= 100 cm^2^ area | 43 (86)  0  0  0 | 29 (80.6)  1 (2.8)  0  0 | 168 (80)  13 (6.2)  7 (3.3)  2 (1) | 2379 (88)  160 (5.9)  133 (4.9)  32 (1.2) | 28 (77.7)  0  1 (2.7)  0 | 40 (80)  1 (2)  0  1 (1) |

Table S3 – Maximum local reactogenicity summary with the onset date within 72 hours following vaccination.

| SYSTEMIC REACTOGENICITY | HVTN 120 (MF59)  (n=50) | HVTN 107 (MF59)  (n=36) | HVTN 100  (MF59)  (n=210) | HVTN 702  (MF59)  (n=2704) | HVTN 107 (Alum)  (n=36) | HVTN 120 (AS01b)  (n=50) |
| --- | --- | --- | --- | --- | --- | --- |
| Malaise/Fatigue (%)  None  Mild  Moderate  Severe | 26 (52)  17 (34)  6 (12)  1 (2) | 32 (88.9)  4 (11.1)  -  - | 121 (57.6)  74 (35.2)  15 (7.1)  - | 2464 (91.1)  218 (8.1)  21 (0.8)  1 (0) | 28 (77.8)  7 (19.4)  1 (2.8)  - | 27 (54)  7 (14)  13 (26)  3 (6) |
| Myalgia (%)  None  Mild  Moderate  Severe | 38 (76)  11 (22)  1 (2)  - | 36 (100)  -  -  - | 124 (59)  64 (30.5)  22 (10.5)  - | 2604 (96.3)  85 (3.1)  15 (0.6)  - | 35 (97.2)  1 (2.8)  -  - | 32 (64)  7 (14)  9 (18)  2 (4) |
| Headache (%)  None  Mild  Moderate  Severe | 30 (60)  15 (30)  5 (10)  - | 28 (77.8)  8 (22.2)  -  - | 130 (61.9)  62 (29.5)  17 (8.1)  1 (0.5) | 2267 (83.8)  317 (11.7)  117 (4.3)  3 (0.1) | 28 (77.8)  6 (16.7)  2 (5.6)  - | 26 (52)  13 (26)  11 (22)  - |
| Nausea (%)  None  Mild  Moderate  Severe | 42 (84)  6 (12)  2 (4)  - | 30 (83.3)  6 (16.7)  -  - | 174 (82.9)  34 (16.2)  2 (1)  - | 2585 (95.6)  104 (3.8)  15 (0.6)  - | 34 (94.4)  2 (5.6)  -  - | 39 (78)  10 (20)  1 (2)  - |
| Vomiting (%)  None  Mild  Moderate  Severe | 48 (96)  1 (2)  1 (2)  - | 36 (100)  -  -  - | 200 (95.2)  9 (4.3)  1 (0.5)  - | 2625 (97.1)  64 (2.4)  15 (0.6)  - | 35 (97.2)  1 (2.8)  -  - | 48 (96)  2 (4)  -  - |
| Chills (%)  None  Mild  Moderate  Severe | 42 (84)  7 (12)  1 (2)  - | 36 (100)  -  -  - | 187 (89)  21 (10)  2 (1)  - | 2614 (96.7)  87 (3.2)  3 (0.1)  - | 34 (94.4)  2 (5.6)  -  - | 33 (66)  10 (20)  6 (12)  1 (2) |
| Arthralgia (%)  None  Mild  Moderate  Severe | 40 (80)  7 (14)  3 (6)  - | 34 (94.4)  2 (5.6)  -  - | 143 (68.1)  57 (27.1)  8 (3.6)  2 (1) | 2623 (97)  74 (2.7)  7 (0.3)  - | 34 (94.4)  2 (5.6)  -  - | 39 (78)  3 (6)  7 (14)  1 (2) |
| Temperature © (%)  < 37.9  38-38.5  38.6-39.2  39.3-39.9  >=40 | 47 (94)  2 (4)  1 (2)  -  - | 36 (100)  -  -  -  - | 192 (91.4)  11 (5.2)  7 (3.3)  -  - | 2409 (89.1)  168 (6.2)  98 (3.6)  19 (0.7)  1 (0) | 31 (86.1)  3 (8.3)  1 (2.8)  -  1 (2.8) | 46 (92)  2 (4)  2 (4)  -  - |

Table S4 – Maximum systemic reactogenicity summary with the onset date within 72 hours following vaccination.

# Figures


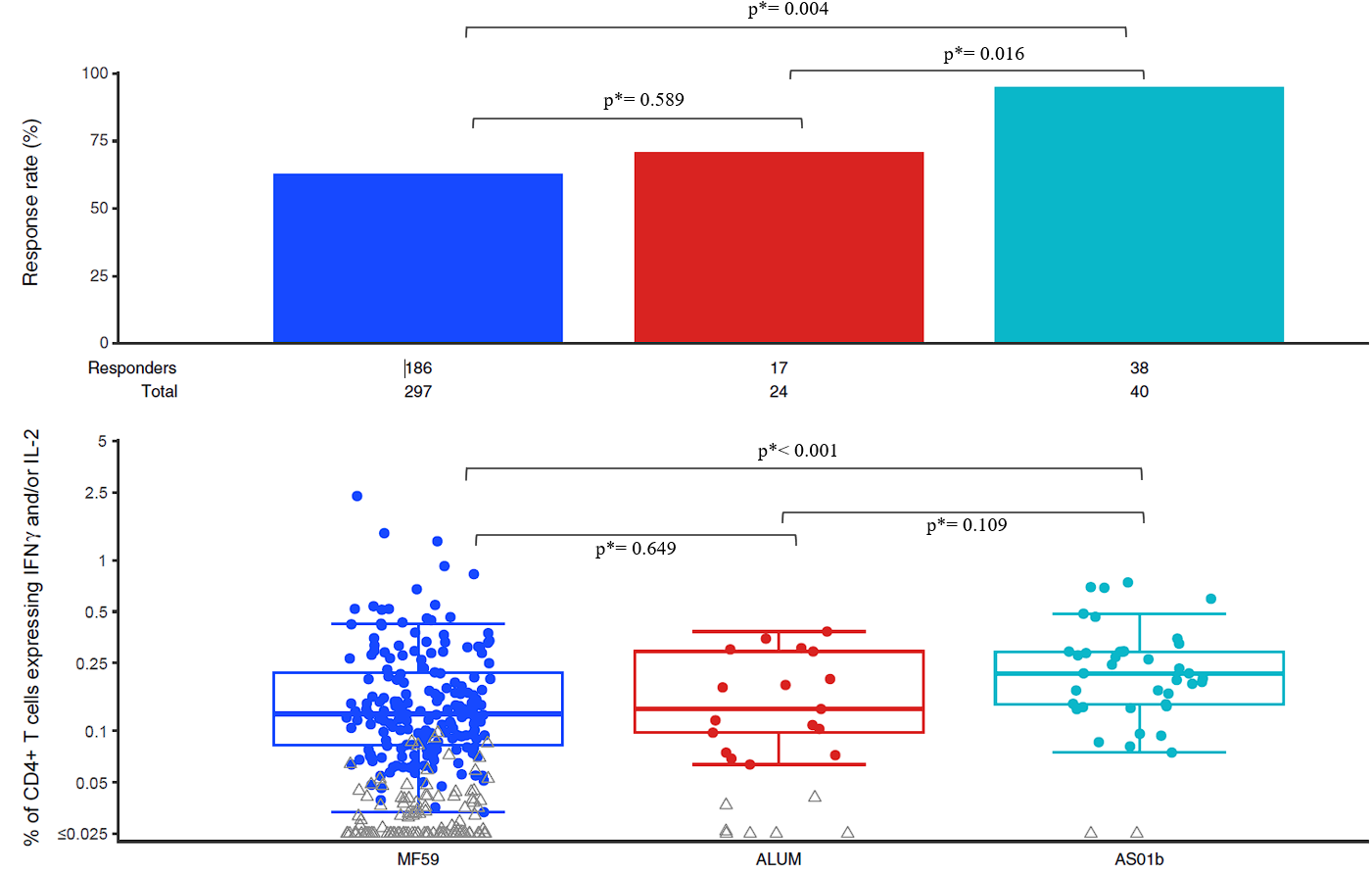


Figure S1- Intracellular cytokine staining (ICS)T-cell assays: Response rates and magnitudes of CD4+ IFNy and/or IL-2 Expression in response to TV1 gp120.


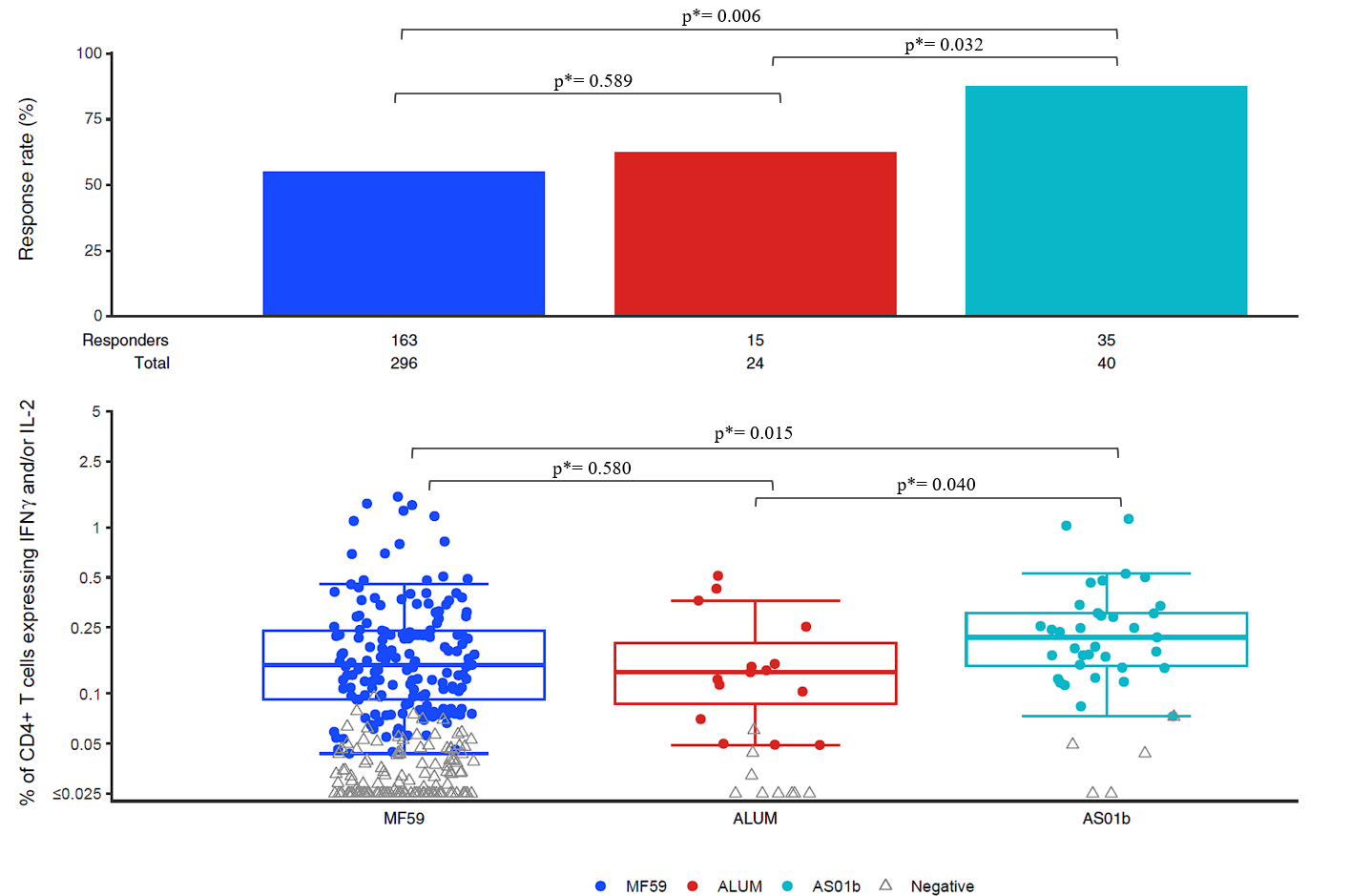


Figure S2- Intracellular cytokine staining (ICS)T-cell assays: Response rates and magnitudes of CD4+ IFNy and/or IL-2 Expression in response to ZM96 gp120.


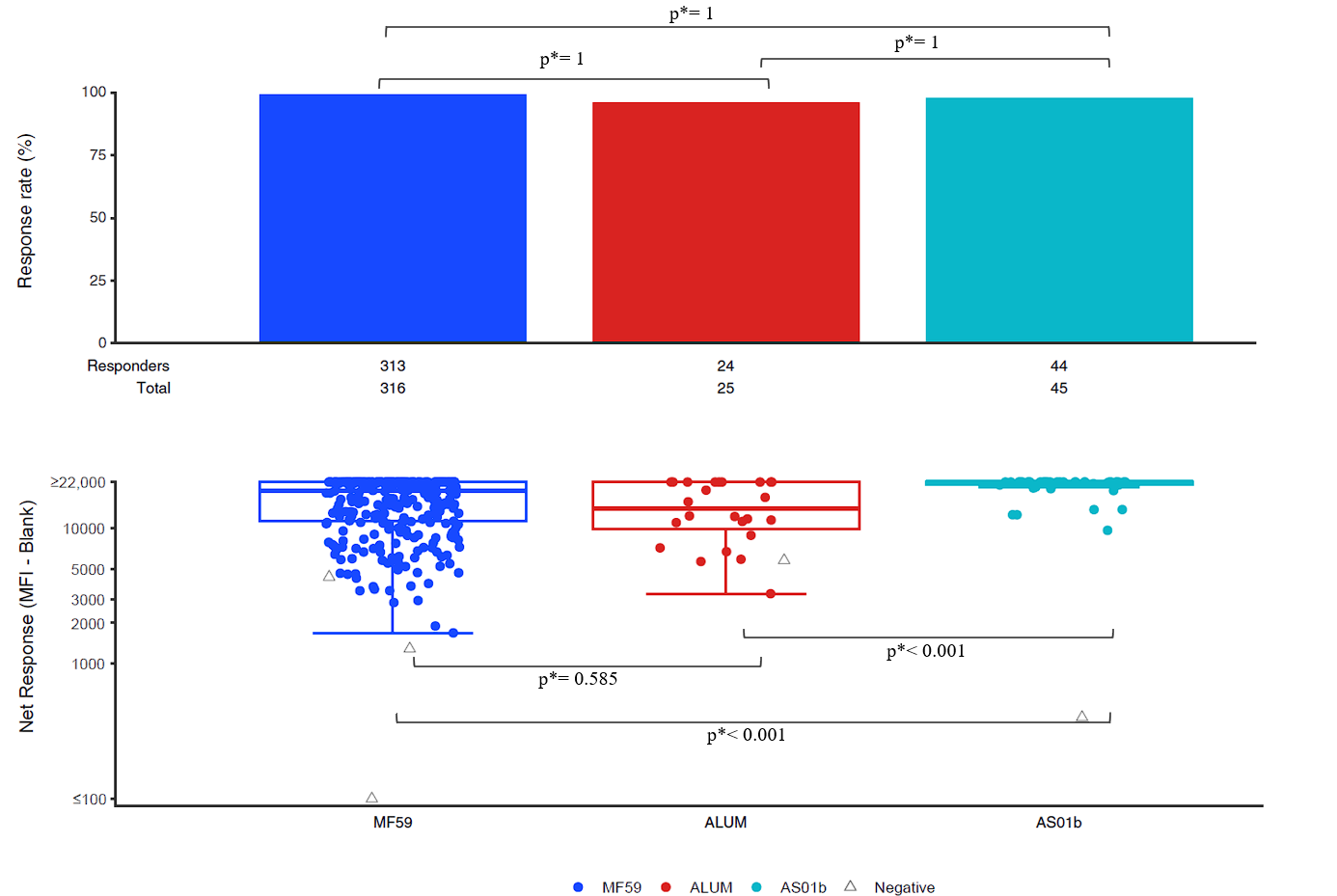


Figure-S3 – IgG Binding Antibody Response rates and magnitudes to con 6 gp 120/B antigen (Dilution 1:50).


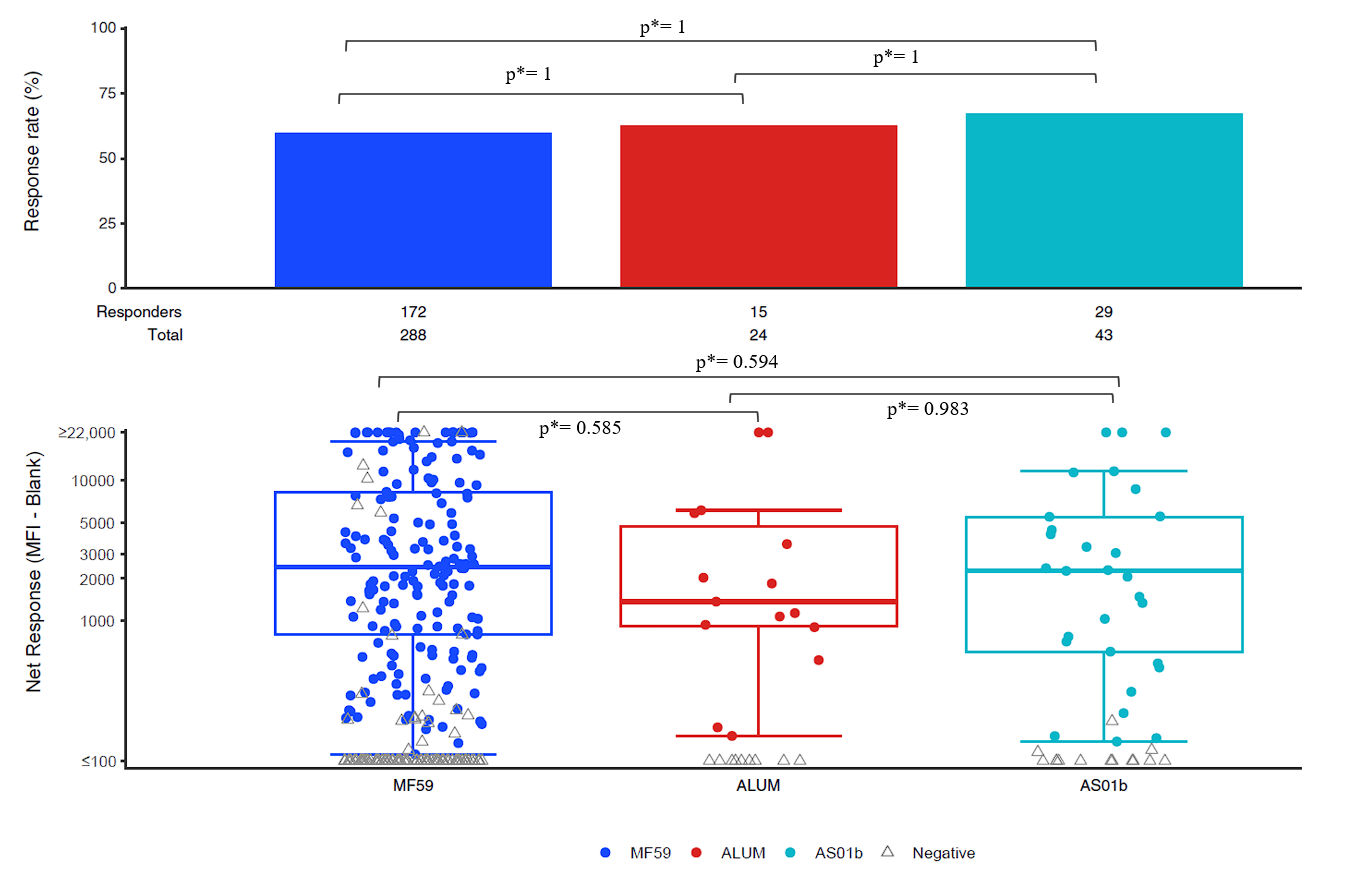


Figure-S4 – IgG Binding Antibody Response rates and magnitudes to gp70-TV1.GSKvacV1V2/293F (Dilution 1:50).


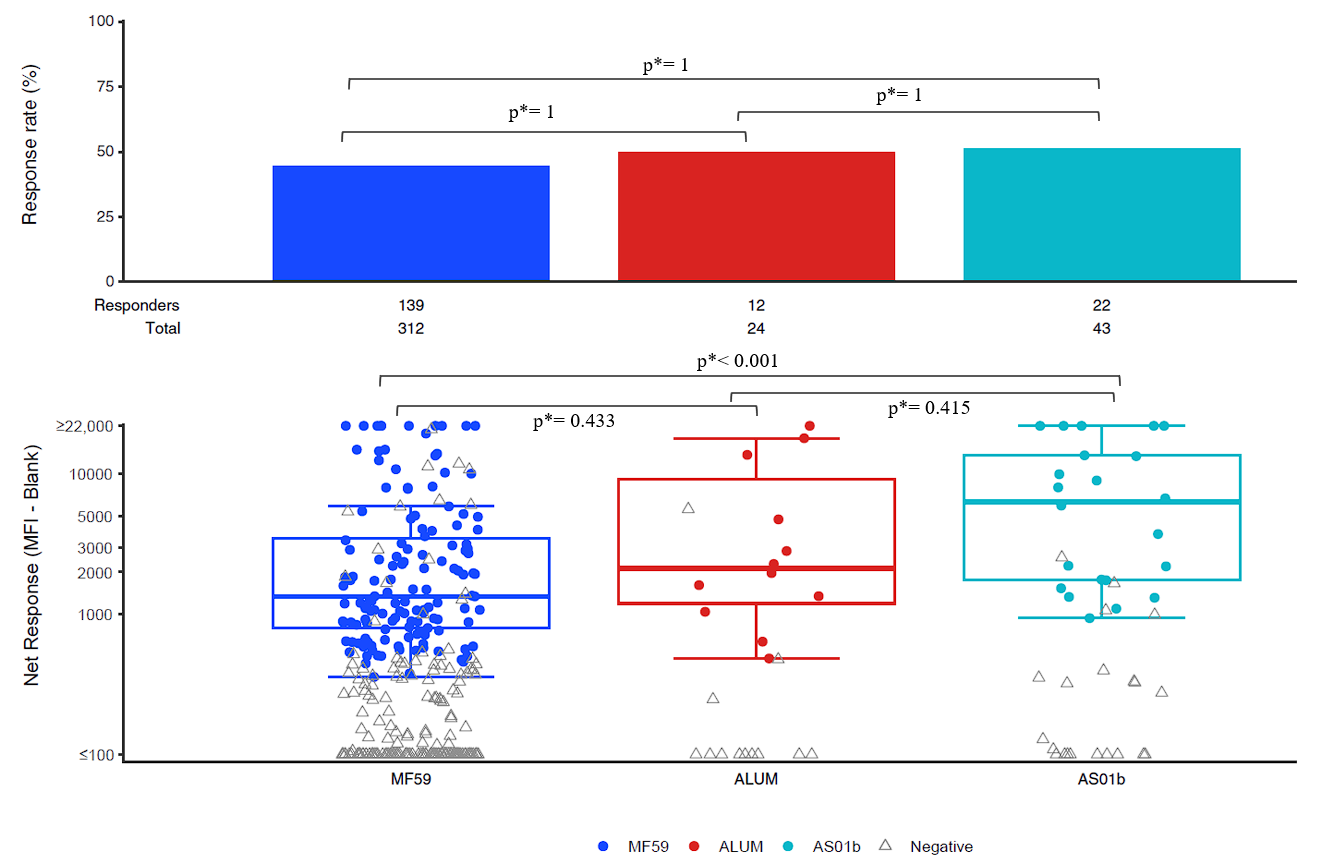


Figure-S5 – IgG Binding Antibody Response rates and magnitudes to gp70-96ZM651.02 V1V2 (Dilution 1:50).


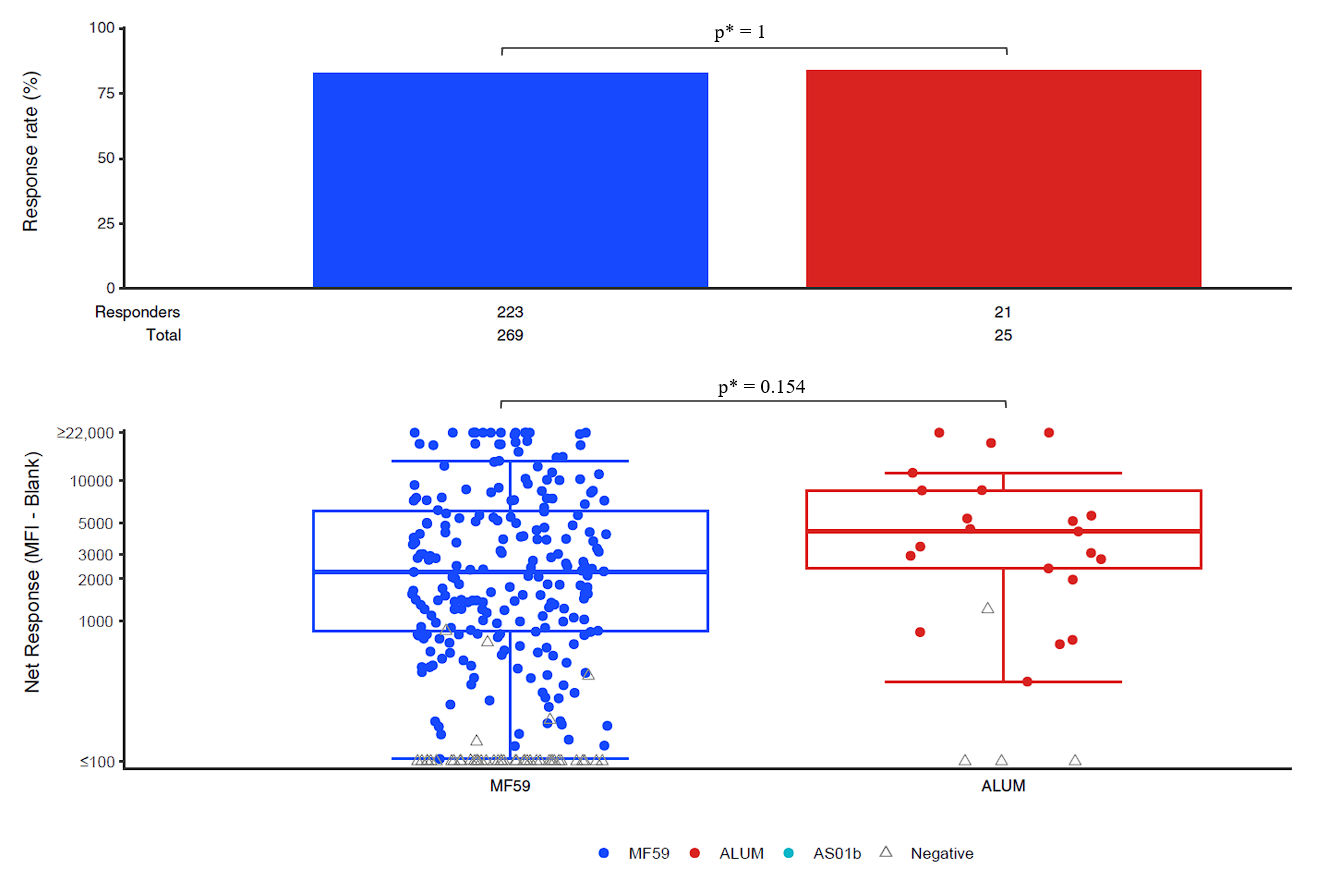


Figure-S6 – IgG Binding Antibody Response rates and magnitudes to AE.A244 V1V2 Tags/293F (Dilution 1:50).


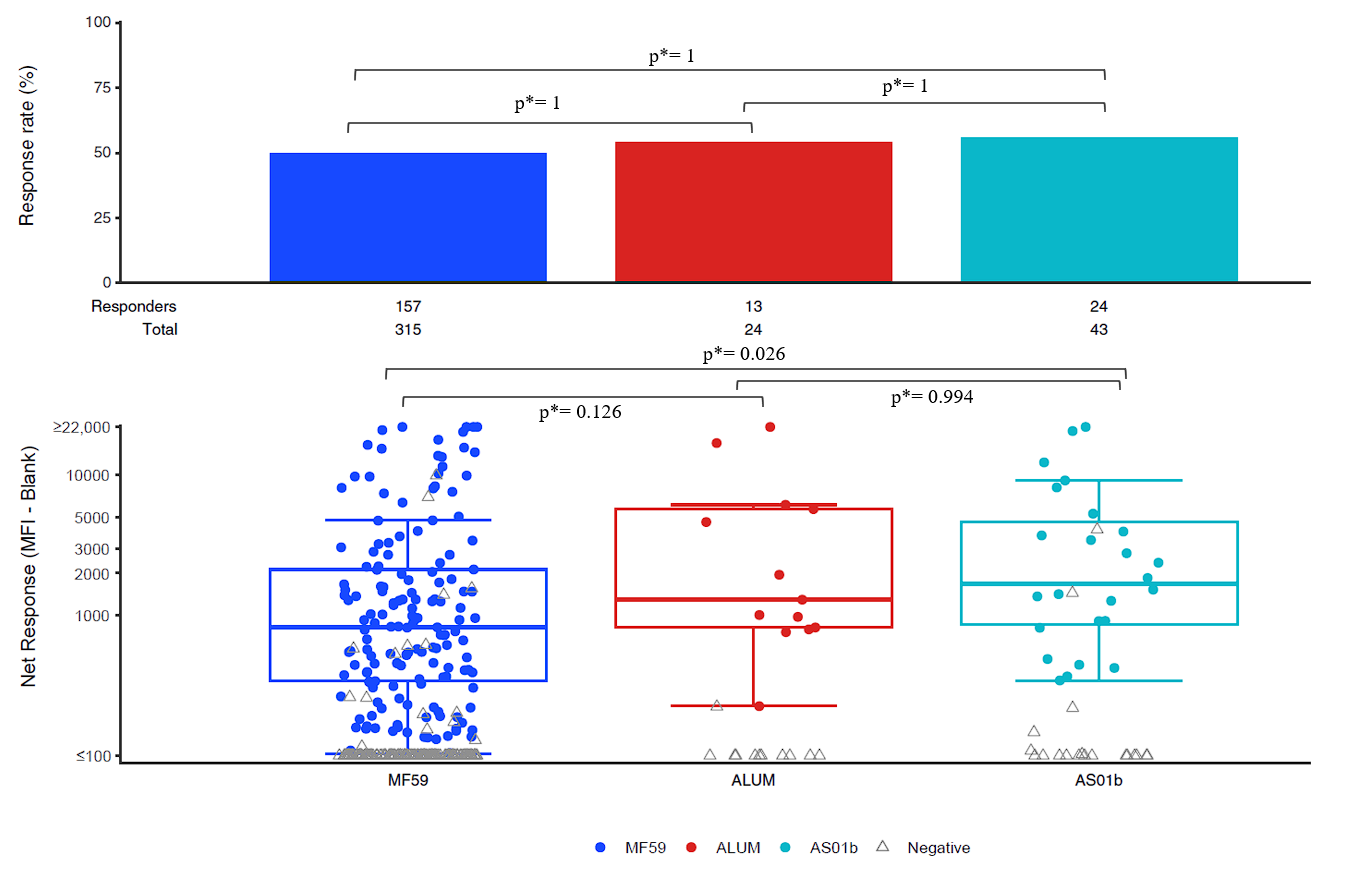


Figure-S7 – IgG Binding Antibody Response rates and magnitudes to gp70_B.CaseA_V1_V2 (Dilution 1:50).
